# Lower extremity movement quality in professional team sport athletes: Inter-rater agreement and relationships with quantitative results from the corresponding pattern

**DOI:** 10.1186/s13102-024-00886-6

**Published:** 2024-04-30

**Authors:** Matthias Keller, Daniel Niederer, René Schwesig, Eduard Kurz

**Affiliations:** 1 OSINSTITUT Ortho & Sport, Munich, Germany; 2 Department of Sports Medicine and Exercise Physiology, Institute of Occupational, Social and Environmental Medicine, Goethe University Frankfurt, Frankfurt, Germany; 3 Department of Orthopedic and Trauma Surgery, Martin-Luther-University Halle-Wittenberg, Ernst-Grube-Str. 40, Halle (Saale), 06120 Germany

**Keywords:** Pre-injury, Screening, Assessment, Movement quality, Rehabilitation, Soccer, LSI, Hop test

## Abstract

**Background:**

Adequate movement control and quality can be prerequisite functions for performance of the lower extremity. The purposes of our work were 1) to explore the agreement of an efficient test battery assessing qualitative movement execution and 2) to determine its consistency with quantitative performance tests from the corresponding movement pattern.

**Methods:**

The participants were professional male association football players competing in the first German Bundesliga. They performed four movement quality tests (Single-limb balance squat, Balance forward hop, Balance side hop, Balance 90° rotation hop) and the corresponding performance tests (Y-balance test, Forward hop for distance, Side hop test, Square hop test). Qualitative tests were judged by two experienced raters; the ratings were compared to determine inter-rater agreement using Kappa statistics. The relationship with the quantitative tests was determined using Spearman’s rank correlations.

**Results:**

Thirty participants (19 to 33 years old) were included in this study. We found an at least substantial level of agreement (Cohen’s Kappa, 0.64-0.84) with an excellent percentage of exact (83-93%) agreement between the two raters for the movement quality tests. Our findings revealed that the quantitative test results are only slightly related to the movement quality outcomes (ρ(27) <|0.3| and *P* > 0.2).

**Conclusions:**

Consequently, the qualitative test results provide unique information and complement corresponding quantitative performance tests in professional football athletes. Their observational judgement of foot position, lower limb alignment and upper body control in sagittal, frontal, and transverse planes is agreeable.

**Supplementary Information:**

The online version contains supplementary material available at 10.1186/s13102-024-00886-6.

## Background

The assessment of movement patterns has an important role for team sport athletes. In the rehabilitation and return-to-sport process, both performance and movement quality of functional tasks like hop for distance tests should be examined as a return-to-sport criterion [1]. More specifically, assessing only distance as the outcome of forward hops is insufficient to detect knee functional movement deficits after anterior cruciate ligament reconstruction [2]. Generally, dynamic jumping, hopping, and cutting maneuvers are frequently used as return-to-sport clearance tests [3]. Typically the outcome is quantitative (such as jumping distance or height). In contrast, qualitative assessments, also of such jumps and hops, are only sparsely adopted. Using a more holistic approach by combining qualitative and quantitative ratings of simple clinical tests, dynamic strength and hop tests is likely to be a more promising way to detect (and subsequently target) functional deficits.

Focusing on the lower extremities, numerous movement quality assessments exist, in particular in athletic team sport settings [4]. Their complexity ranges from simple (mirroring activities of daily living) to highly demanding (including fast turns and stops with jumps and cutting maneuvers). Those adopted are the single-limb squat [5] or the lateral step-down test [6] from the simple category and the landing error scoring system [7] and single-limb drop jumps from the more demanding categories [8]. All these tests share sufficient to excellent reliability and video-based classifications of estimated dynamic changes of frontal and sagittal plane projection angles as rating criteria.

For injury risk profiling or to identify potentially improvable movement patterns, hop tests mirroring progressive movement complexities, are mostly used. The increasing demands can be categorized into (four) levels (return-to-activity algorithm, RTAA). Level I: simple everyday movements through = Single-limb balance squat; level II: dynamic movements without = Balance forward hop; level III: such with lateral movements and (simple) twisting movements = Balance side hop; and level IV: multidirectional cutting maneuvers = Balance 90° rotation hop [9]. Qualitative video-based test batteries mirroring this whole movement complexity spectrum are used in athletic/clinical settings, although not ubiquitously [9]. However, their inter-rater agreement is unknown. Despite a theoretical founding, it is also unknown whether these qualitative tests deliver complementary information to commonly used quantitative hop tests.

In terms of movement complexity, the corresponding quantitative hop tests are well validated and are frequently used in clinical or athletic test settings [10, 11]. For the complexity levels I and II, the Y-balance and the forward hop for distance are frequently adopted in functional lower extremity evaluations, with standardized (classic or rebound) Side [12, 13] and Square hops frequently used for the levels III and IV. Generally, performing differentiated test batteries instead of single tests is likely to produce more valid results [14].

As the reliability of video-based assessments and ratings of the motor complexity mirroring lower extremity functional tests Single-limb balance squat test, Balance forward hop test, Balance side hop test, and Balance 90° rotation hop test and their relationships with classical tests are as yet unknown, the purpose of our work was to provide evidence on 1) the inter-rater agreement of four movement quality tests (Single-limb balance squat—Balance forward hop—Balance side hop—Balance 90° rotation hop) and 2) determine the potential relationships of these tests with the results of four quantitative tests assessing the corresponding movement pattern. We hypothesized that all movement quality tests (1) show sufficient inter-rater agreement and (2) display unique (functional movement characteristic) information.

## Methods

This study is reported following the STROBE guidelines [15].

### Participants

Thirty professional male association football players volunteered in this cross-sectional diagnosis study. All participants belonged to the top team of a football club competing in the first German Bundesliga at the 2017/18 season.

Exclusion criteria were any acute medical history and/or previous surgery of the lower extremities. According to the Declaration of Helsinki, data collection was conducted after written informed consent was obtained from the volunteers. The Institutional Review Board of the Martin Luther University of Halle-Wittenberg (reference number: 2013-13) approved the study protocol. The tests were part of the routinely conducted pre-season indoor screening process, containing qualitative and quantitative capacities of the lower extremity. All quantitative outcomes are measured with an SI unit (interval scale). Qualitative outcomes, in contrast, are rated subjectively (ordinal scale).

### Procedures

**Fig. 1:**
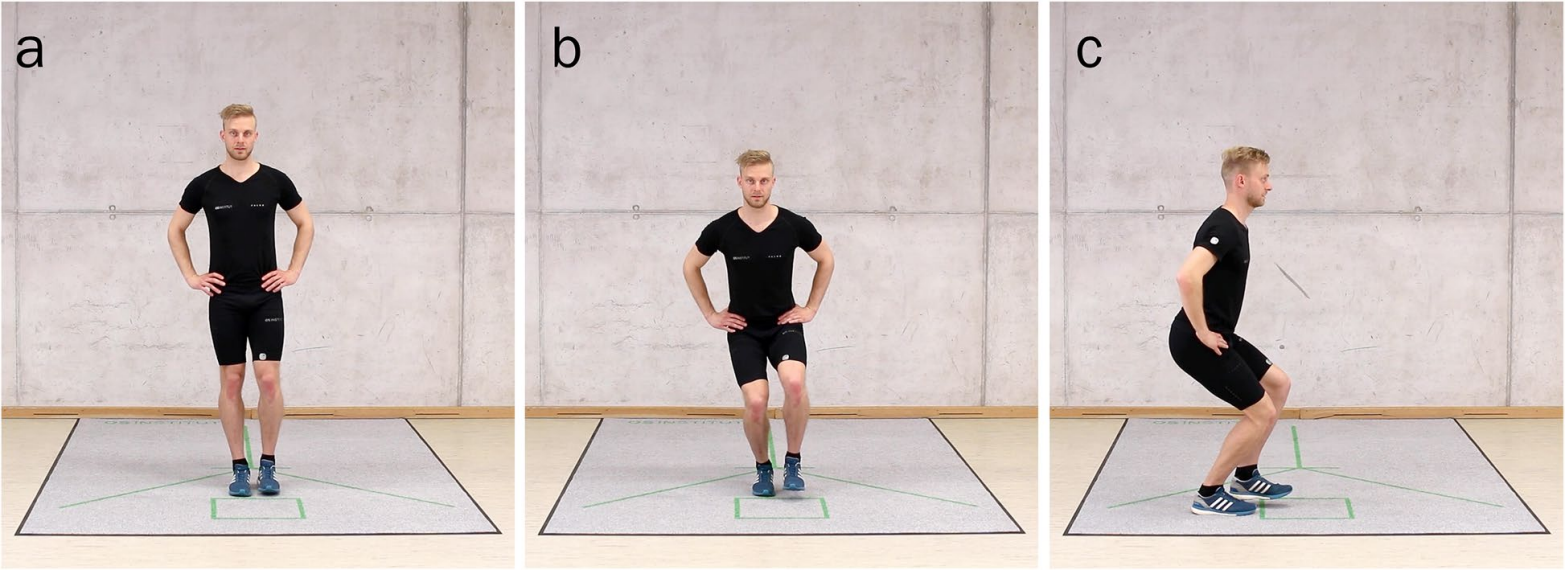
Single-limb balance squat. **a** Starting position: standing on one lower limb (here: right) with the unloaded lower limb (here: left) slightly above the floor and with arms akimbo. **b** Key observations from the frontal view include controlled alignment of the lower limb joints and upright trunk without lateral shift. **c** From the lateral view, heels flat, upright trunk, flexion movements at the ankle, knee, and hip joints

**Fig. 2:**
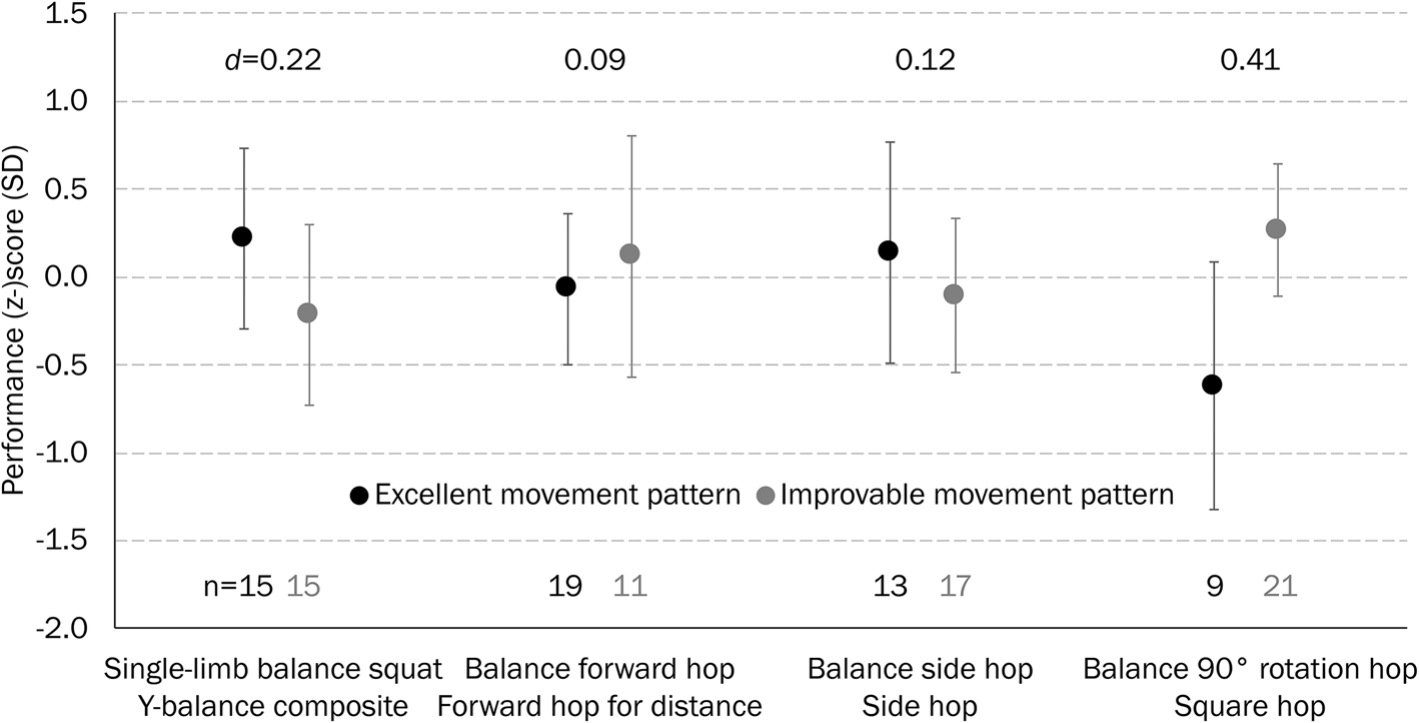
Movement quality in subgroups of athletes presented a good (score = 5) or poor (score < 5) pattern with the corresponding quantity test performance (z-score) for the dominant lower limb. Values are means with 95% confidence intervals

The test battery (RTAA) performed consisted of eight unilaterally performed tests [9]. Four tests comprising quantitative performance were carried out: 1) Y-balance test, 2) Forward hop for distance, 3) Side hop, and 4) Square hop tests. Each of the quantitative tests was preceded by a qualitative test from the corresponding movement pattern: 1) Single-limb balance squat (see Fig. 1) and 2) Balance forward hop, 3) Balance side hop, and 4) Balance 90° rotation hop over a distance of approximately 40 cm. All tests were executed with participants wearing shoes, with hands akimbo for the movement quality tests only.

Each of the movement quality tests was assessed visually by rating five different segment-specific postural orientations modified according Nae et al. [16] after concurrently videotaped frontal and sagittal views of two executed trials (Table 1). Scoring was undertaken dichotomously for exactly (= 5) or erroneously (< 5) performed postural orientations on the better-executed trial. Further, all quality scores were summed up for each test pattern (both sides, range 0 to 10), respectively. The rating was performed by two experienced (> 12 years) physiotherapists independently. Only movements with a total score of five were considered as a qualitatively excellent pattern. Movements with a total score of four or lower were considered as a qualitatively improvable pattern.

**Table 1:** Overview of the criteria applied for judging the quality of the movement patterns (adopted from Keller et al. [9])

**Criteria**		**Frontal plane**	**Sagittal plane**
Foot position	1	Adequate foot placement, whole sole supported, absence of foot pronation	
Knee alignment	2 and 3	Appropriate medial/ lateral position control	Adequate knee/ hip flexion
Trunk control	4 and 5	No lateral trunk motion	Aligned parallel to lower leg, no excessive trunk flexion

After completing the (originally equipped) Y balance test (detailed in Plisky et al. [17]), participants performed three attempts of the forward hop test for distance on each side. The distance reached, from toe to heel, was recorded in centimeters. Only successfully completed forward hops for distance (safe and stable landing, without losing balance) were considered for further analysis. For the forward hop distances or the Y-balance reach distances, the highest values achieved (best of three) were used for further analyses.

After short (10 s) familiarizations with the requested rebound actions, the Side hop and Square hop tests were performed for 30 s over a 40 × 40 cm square (line width 2 cm). Here, a modified version of the previously described Square hop test [15, 18] was used. The complete Side and Square hop tests were performed once for each side and videotaped (four files for each participant). This enabled an exact analysis of the total contacts and faults. All files were analyzed offline by the same investigator (MK). The number of faults was subtracted from the total contacts, resulting in regular contacts [14].

### Outcomes

Athletes’ dominant lower limb (i.e., limb preferred to kick a ball) was determined. The length of the participant’s right lower limb (distance from the anterior superior iliac spine to the medial malleolus) was measured for normalization purposes.

Limb symmetry indices (LSI) were calculated from absolute values of the dominant and non-dominant sides: Values of the worse side were multiplied by 100 and divided by those of the better side. Values between 90 and 100% correspond to the range of physiological variability, which in turn designate symmetry between sides. To compare the performance between the different tests, the results of the quantitative tests were converted into z-scores. The z-scores indicate how many standard deviations an athlete’s score is away from the sample mean.

### Sample size estimation

Sample size estimation was done based on the recommendations of Shoukri et al. [19]. Assuming an expected Kappa of 0.6, with an imprecision of 0.3, a tolerable alpha error of 5% and beta error of 20%, 28 participants need to be analyzed.

### Statistics

Statistical analyses were carried out using the SPSS 28.0 (IBM Corp., Armonk, NY, USA) software package. Data distribution of the outcome variables was checked visually and using the Shapiro-Wilk test.

Descriptively, the participant characteristics are presented as mean, standard deviation (SD) with minimum and maximum data values, while results in the figures are provided as means with 95% confidence intervals (CI).

To determine inter-rater agreement, Kappa statistic and percentage agreement was calculated for all movement quality tests (inter-observer agreement). For nominal (dichotomous scoring) or ordinal (summed up quality scores) outcomes, Cohen’s Kappa [18] or Weighted Kappa [20] coefficients were used. As suggested by Viera and Garrett [21] Kappa values < 0.20, 0.21-0.40, 0.41-0.60, 0.61-0.80, 0.81-1.00 indicated trivial, fair, moderate, substantial, or nearly perfect agreement, respectively. Percentage agreement was calculated based on the absolute (number of times raters agree) agreement divided by the number of athletes.

To reveal a potential relationship with the corresponding quantitative tests, correlations between the different interval scaled outcome measures were examined using Pearson’s correlations: Associations between movement quality scores and quantitative performance tests were examined using non-parametric partial (Spearman’s rank) correlations, controlling for side.

As explorative analyses, first, comparisons between the values of the better and worse limbs were performed using the Student’s t test for paired samples. Effect sizes between the limbs were calculated using Cohen’s *d* with values > 0.2, > 0.5, or > 0.8 indicating small, moderate, or large effects, respectively. As further secondary comparisons, differences between the excellent pattern and improvable pattern participants (subgroups) were analyzed using Student’s unpaired t tests of the quantitative test results only.

## Results

### Participants

All participants performed all measurements, no one withdrew consent, and no participant had to be excluded. The participants were on average 25.6 years old (minimum-maximum: 19-33). For the anthropometric data of the athletes included, please refer to Table 2. In 87% of the participants the right lower limb was their dominant lower limb, with no participant presenting dual dominance.

**Table 2:** Anthropometric characteristics of study participants (*n* = 30)

**Variables**		**Mean (standard deviation)**		**Minimum to maximum**
Body height	[m]	1.84	(0.06)	1.75 to 1.96
Body mass	[kg]	80.8	(5.9)	66.5 to 92.6
Body mass index	[kg/m^2^]	23.81	(0.96)	21.71 to 25.59
Lower limb length	[m]	0.99	(0.05)	0.91 to 1.10

### Movement quality ratings

The ratings of the reference (rater 1) revealed 4.5 (4.0 to 5.0, median with 1st to 3rd quartiles), 5.0 (4.0 to 5.0), 4.0 (4.0 to 5.0), and 4.0 (3.0 to 5.0) for the Single-limb balance squat, the Balance forward hop, the Balance side hop, and the Balance 90° rotation hop, respectively. Correspondingly, 15, 19, 13, and 9 out of 30 athletes showed an excellent pattern on the movement quality tests on their dominant side (Fig. 2).

### Performance results

On average the direction corrected LSI values of all quantitative tests were above 90%. For the characteristics without landing impact the proportion of athletes who performed worse on their dominant side was between 0.3 and 0.5, whereas for the tests with impact (hop tests) there was a greater range (0.3-0.8). The results of the quantitative performance tests are displayed in Table 3.

**Table 3:** Absolute and relative results of the different Y-balance test directions and hop tests with effect sizes (ES) and LSI values. Values presented as mean (standard deviation, minimum to maximum)

**Test or direction**		**Dominant**	**Non-dominant**	**ES**	**LSI [%]**
Anterior	[cm]	71 (7, 54 to 83)	72 (7, 58 to 85)	0.07	96 (4, 82 to 100)
	[%LLL]	72 (6, 56 to 81)	73 (6, 57 to 84)	0.08	
Postero-medial	[cm]	113 (10, 86 to 134)	114 (9, 90 to 135)	0.21	97 (3, 89 to 100)
	[%LLL]	114 (8, 91 to 127)	115 (7, 96 to 128)	0.22	
Postero-lateral	[cm]	112 (9, 96 to 134)	110 (8, 98 to 134)	0.44	97 (3, 92 to 100)
	[%LLL]	113 (7, 97 to 125)	111 (6, 101 to 122)	0.43	
Composite	[cm]	294 (22, 262 to 347)	295 (20, 260 to 348)	0.10	98 (2, 95 to 100)
	[%LLL]	100 (5, 90 to 109)	99 (5, 87 to 109)	0.09	
Forward hop	[cm]	219 (16, 179 to 251)	225 (17, 187 to 267)	0.43	95 (3, 89 to 100)
	[%LLL]	222 (17, 184 to 247)	228 (19, 195 to 259)	0.44	
Side hop		68 (7, 51 to 84)	67 (7, 52 to 81)	0.21	94 (5, 82 to 100)
Square hop		83 (13, 47 to 107)	81 (13, 50 to 115)	0.21	92 (8, 68 to 100)

Normalized distances (reach distance divided by the participant’s LLL multiplied by 100); *LLL* lower limb length (anterior superior iliac spine – medial malleolus); Composite distance: sum of all three reach directions (anterior, posteromedial, and posterolateral); Composite score: relative measure of all three directions (anterior, posteromedial, and posterolateral) divided by three times the participant’s LLL and multiplied by 100; Side and Square hop: only regular contacts (total minus faults) within 30 s are considered; LSI, direction corrected (worse divided by better side) limb symmetry index

### Degree of inter-rater agreement

A substantial level of agreement was found between the two raters (*P* < 0.001). Cohen’s Kappa values were between 0.64 (substantial) and 0.84 (almost perfect), and the percentage of exact agreement between examiners was excellent (83-93%, Table 4). More than 57% of the summed-up scores for both sides showed no difference between raters (see Supplement materials 1, 2, 3 and 4). In 8%, a 2-point difference or at most a 3-point difference (*n* = 2/120) was found.

**Table 4:** Absolute and percentage agreement between raters with the respective Kappa values

**Task**	**Absolute agreement**		**Percentage agreement**		**Cohen’s Kappa**	**Weighted Kappa**
	Dominant	Non-dominant	Dominant	Non-dominant		
Single-limb balance squat	26	27	87	90	0.75	0.80
Balance forward hop	26	25	87	83	0.64	0.59
Balance side hop	27	28	90	93	0.79	0.65
Balance 90° rotation hop	26	28	87	93	0.84	0.61

### Associations between movement quality and performance results

The athletes with an excellent movement quality on the Single-limb balance squat performed on average better on the Y-balance test than their peers with limited movement quality. By contrast, the athletes with limited movement quality on the Balance 90° rotation hop had on average more regular contacts in the Square hop test as compared with those with excellent movement quality.

**Fig. 3:**
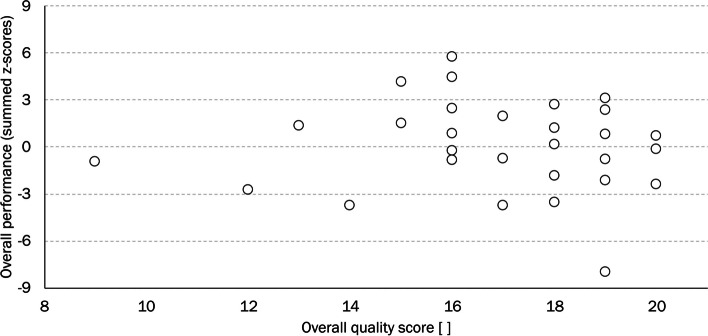
Overall performance of the movement quality scores against the summed-up z-scores of the quantity test performances for the dominant lower limb

No associations were found between the movement quality scores (sum of sides) and the corresponding performance tests (sum of sides), while controlling for the dominant or non-dominant limb (all ρ(27) <|0.3| and *P* > 0.2). The overall performance of the movement quality scores against the summed-up z-scores of the performance tests for the dominant lower limb are depicted in Fig. 3.

## Discussion

This investigation primarily tested the inter-rater agreement of video-based movement quality ratings of different motor complexity mirroring lower extremity functional tests in top-level association football players. Secondly, the tests’ agreement with classical quantitative test results was targeted. For each of the qualitative test outcomes, we found at least a substantial level of reliability and an excellent percentage of exact agreement between the two experienced raters. Athletes’ movement quality was not related to performance in either type of quantitative test.

Previous investigations on visual assessments of movement quality of the lower extremity reported moderate to good inter-rater reliability coefficients and are therefore in line with our findings. In particular, tests without body transitions (single-limb mini-squat, Kappa = 0.92 [22]) in healthy individuals were found to show better results compared with transitional tests (lateral step-down in healthy Kappa = 0.59 or participants with patellofemoral pain, Kappa = 0.67 [23]). The lower Kappa value during the single-limb balance squat in our study most probably results from the number of criteria to be judged. In the study by Ageberg et al. [22], solely the knee position in relation to the foot was examined. In addition, their test was performed barefoot. A deficient movement quality on the single-limb squat was found in 33 patients six months after ACL reconstruction [24]. Patients with an inadequate quality of the single-limb squat showed lower hip abduction strength and forward hop for distance. With the exception of the balance forward hop, the transitional quality tests in this study revealed almost perfect inter-observer agreement. Our findings suggest that the higher the control demand, the more consistent the rater’s evaluation.

Altered movement patterns may limit performance and potentially enhance the risk of non-contact injuries. In his review on late-stage rehabilitation after anterior cruciate ligament reconstruction, Buckthorpe [25] identifies movement quality deficits as one factor associated with re-injury risk. Further, recent studies showed that judging the quality of single-limb landings may provide valuable information for deciding on successful rehabilitation after ACL reconstruction surgery (1). Moreover, judging the ability or inability to perform required movements or sustain requested positions enhances the practicability (cf. Ekegren et al. [26]). However, movement quality assessment is not included by default and often costly and time consuming. We observed in practice that athletes’ movement quality was mostly not related to performance in either type of quantitative test. The qualitative test results consequently provide unique information and complement corresponding performance tests. Because the importance and relevance of movement quality testing is also proven [1, 25], qualitative tests are of great relevance, especially in rehabilitation settings.

A recently published systematic review on the lower quarter Y-balance test included 57 studies in total and 18 (32%) on association football players in general [27]. Butler et al. [28] examined 44 professional players from the United States and Germany. They found Y-balance composite scores comparable to ours (102%), though averaged between sides. In contrast, Lopez-Valenciano et al. [29] revealed, in a well comparable sample (*n* = 88), an average Y-balance composite score of 88 or 89% for the dominant or non-dominant lower limb, respectively. Their averaged results correspond to our minimum values. This is most likely attributed to the different execution. Despite the fact that the athletes were wearing shoes, and similarly to the hop tests performed, our athletes were allowed to move their arms in a natural fashion [30]. Consequently, the restriction of arm movement will lead to a loss of about 10% of the composite reach distance.

Delvaux and colleagues [31] examined the forward hop for distance in 38 professional football players of comparable age, stature, and execution. Their athletes demonstrated greater average forward hop distances (dominant: 234 cm, non-dominant: 240 cm) as compared with ours. However, their measurements were corrupted by the measurement methodology. They reported the distance measured from toe to toe. This incorporates the participant’s foot length and consequently a further hop distance (cf. Read et al. [32]). Interestingly, in both cohorts the non-dominant lower limb revealed a better performance of relevant magnitude. In another study on sub-elite football players (*n* = 10), the average forward hop distance was 188 cm [33], which is far below the results reported in elite players.

To quantify hop performance in the frontal plane, three different Side hop tests are used. While the rebound Side hop [13] is normalized to participants’ body height, the repetitive Side hop tests use defined distances of 30 cm [34] or 40 cm [14]. The 30 cm Side hop is conducted within 10 s. Due to the smaller distance to hop over, shorter ground contact times are to be expected. By contrast, the 40 cm Side hop lasts 30 s and includes an endurance component. Good to excellent inter-session reliability of the 40 cm Side hop was demonstrated by healthy male and female athletes [12]. Those athletes arrived on average at 47 regular contacts, which is lower than the minimum values of our participants. These marked differences are explained either by the lower competitive level, the execution (hands on hips) or both. A football match was shown to influence the number of Side hop contacts in injured amateur football players returning to performance as well as their uninjured teammates [35]. Although the athletes were allowed to use their arms during the Side hop, they underperformed our participants by on average 14 contacts.

Like the 30 cm Side hop, the Square hop is mainly forefoot dominant along with potentially shorter ground contact times. Two studies were identified that incorporated a modified version of the Square hop test in football players [33, 36]. In the study of Ros and colleagues [33], sub-elite football players performed the Square hop test once before and three times after the Yo-Yo Intermittent Recovery test. The participants showed on average 26 contacts before the endurance test, which increased up to 30 during recovery [33]. Östenberg et al. [36] examined 101 female football players. They found on average 21 valid contacts. In both aforementioned studies, only the contacts the participant’s foot touched inside the square were calculated. To compare those results with the finding in our study, the numbers need to be multiplied by two. Even then, our results are hardly comparable. The ground contact time of the repetitive hops in our study was shown to be approximately half compared with the results of the two articles mentioned [33, 36], and indicates a different solution to the motor task. Our participants were instructed to achieve as many regular contacts (faults removed) as possible. Thus, the performance aspect was emphasized during the Square hop test. Conversely, in the studies by Östenberg [36] and Ros [33] more time was left for the investigator to assess the movement quality to some extent.

By examining lower limb movement quality (in terms of present substitution patterns) and hop test performance in 53 persons (age: 18 to 35 years) with a moderate to high level of physical activity after two to five years after ACL injury, Trulsson et al. [37] found inverse moderate associations. Since higher values of substitution patterns represent worse neuromuscular control, their findings suggest that limited movement quality will be related to worse performance on hop tests. The results of our investigation failed to establish such a relationship per se. However, an in-depth examination provided more clarity. Subgrouping the participants according to the movement quality presented on their dominant side revealed considerable differences, at least for the Y-balance and Square hop tests. For a closed chain movement, the participants with better movement quality tend to outperform those with worse neuromuscular control. Conversely, in a more demanding task with multiple hops and minimal ground contact time, the quality of movement was unable to predict better performance.

This study comprises some limitations that need to be addressed: In this investigation professional male association football players were included. The results are therefore not necessarily transferable to different team sports or female athletes. Also, both raters were experienced with the observations presented. Whether or not novice raters would obtain comparable results remains unknown. Furthermore, the tests presented were part of the pre-season screening and were conducted indoors. Consequently, conclusions on sport-specific performance are limited.

By implementing simple qualitative and quantitative tests, functional profiles of players and teams can be created during preseason screenings. Although periodic health examinations still fail to provide sufficient data to predict non-contact injuries [38], they are able to mirror adequate conditions of athletes’ exposure to specific demands. Functional profiling as part of pre-season screenings comprises critical findings in case of future injuries. Especially the rehabilitation specialist can use the results as a baseline to better guide the rehabilitation process. Due to limited time schedules for professional team sports, efficient test batteries have become more important. Aspects of movement quality may be captured separately from performance characteristics.

### Supplementary Information

**Additional file 1:** **Figure 1S.** Comparison of raters’ judgements of movement quality of the Balance single-limb squat (sum of both sides).

**Additional file 2:** **Figure 2S.** Comparison of raters’ judgements of movement quality of the Balance forward hop (sum of both sides).

**Additional file 3:** **Figure 3S.** Comparison of raters’ judgements of movement quality of the Balance side hop (sum of both sides).

**Additional file 4:** **Figure 4S.** Comparison of raters’ judgements of movement quality of the Balance 90° rotation hop (sum of both sides).

## Data Availability

The datasets used and analyzed during the current study are available from the corresponding author on reasonable request.
