# Supplementary figures and images for: Lower extremity movement quality in professional team sport athletes: Inter-rater agreement and relationships with quantitative results from the corresponding pattern
Source: BMC Sports Sci Med Rehabil. 2024 Apr 30;16:98. doi: 10.1186/s13102-024-00886-6 (PMC11059726; doi:10.1186/s13102-024-00886-6)

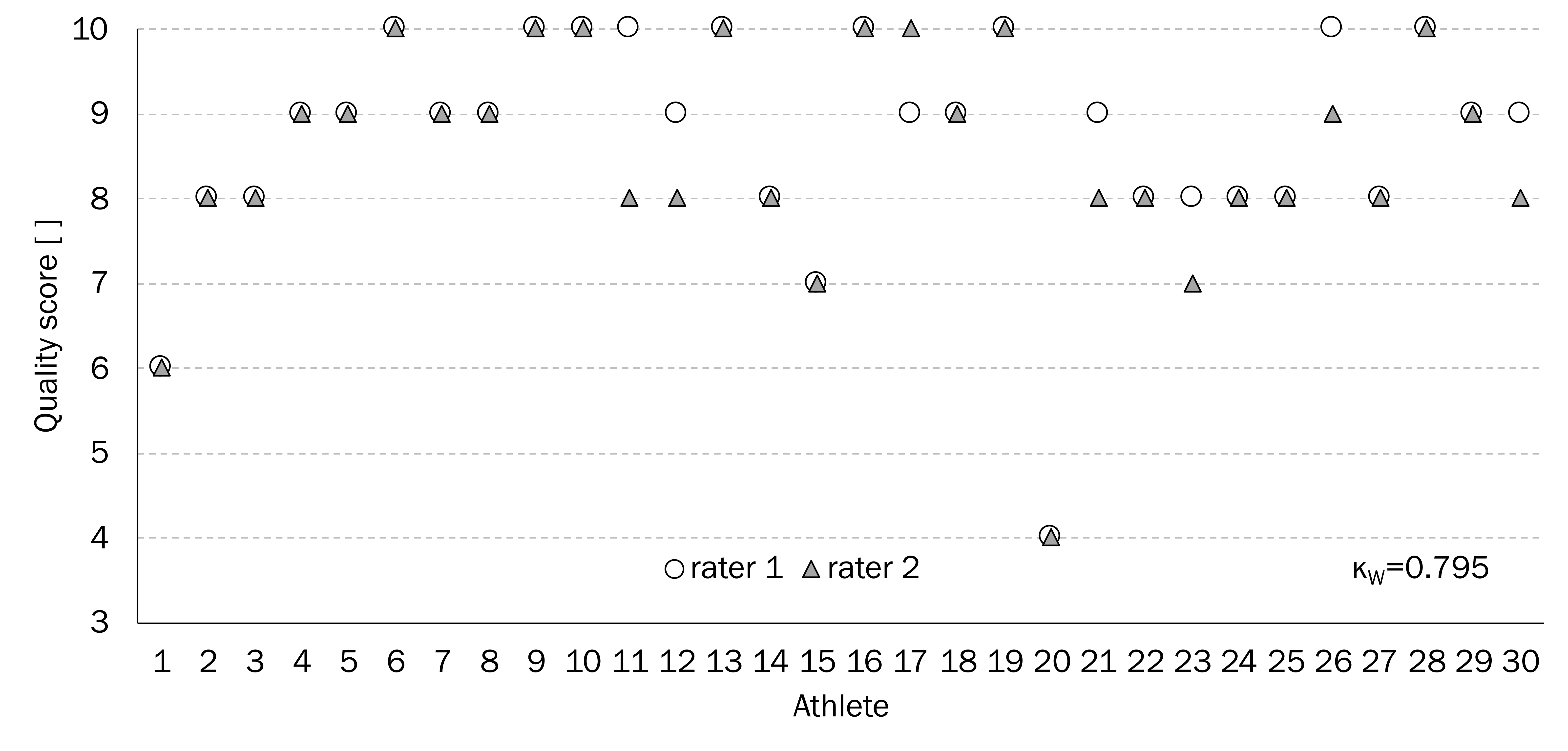

Supplement: Supplementary file 1 — Additional file 1: Figure 1S. Comparison of raters’ judgements of movement quality of the Balance single-limb squat (sum of both sides). [file 13102_2024_886_MOESM1_ESM.png]

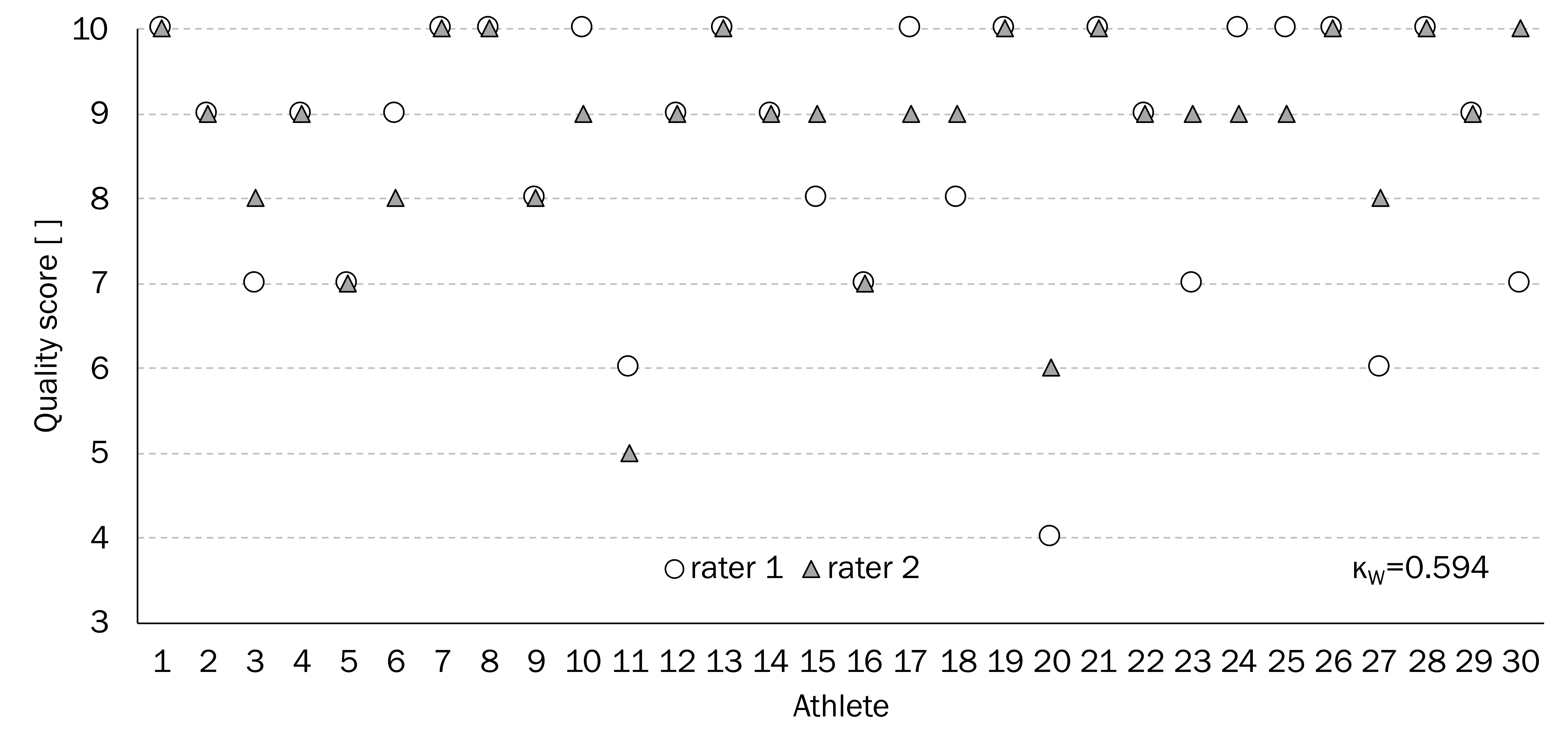

Supplement: Supplementary file 2 — Additional file 2: Figure 2S. Comparison of raters’ judgements of movement quality of the Balance forward hop (sum of both sides). [file 13102_2024_886_MOESM2_ESM.png]

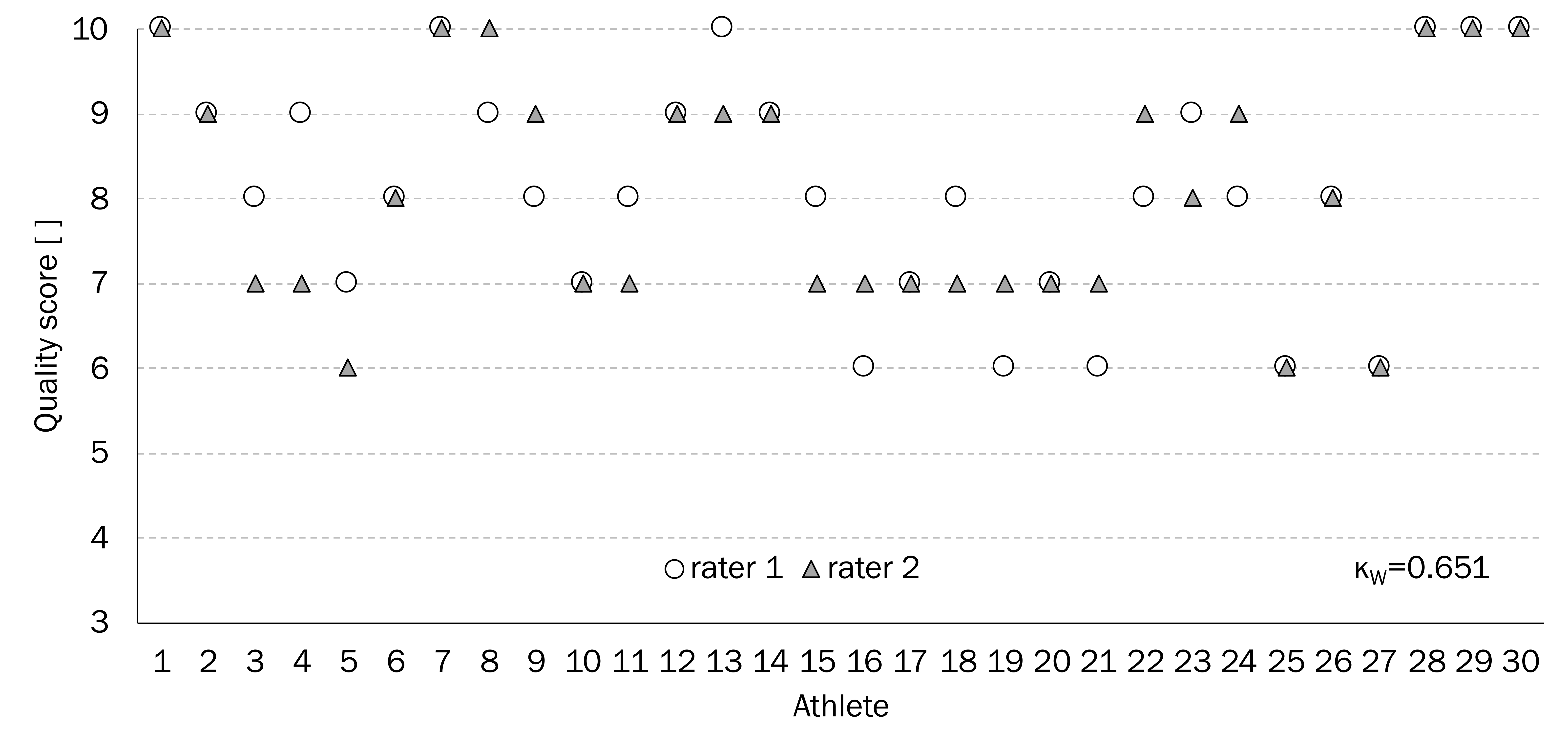

Supplement: Supplementary file 3 — Additional file 3: Figure 3S. Comparison of raters’ judgements of movement quality of the Balance side hop (sum of both sides). [file 13102_2024_886_MOESM3_ESM.png]

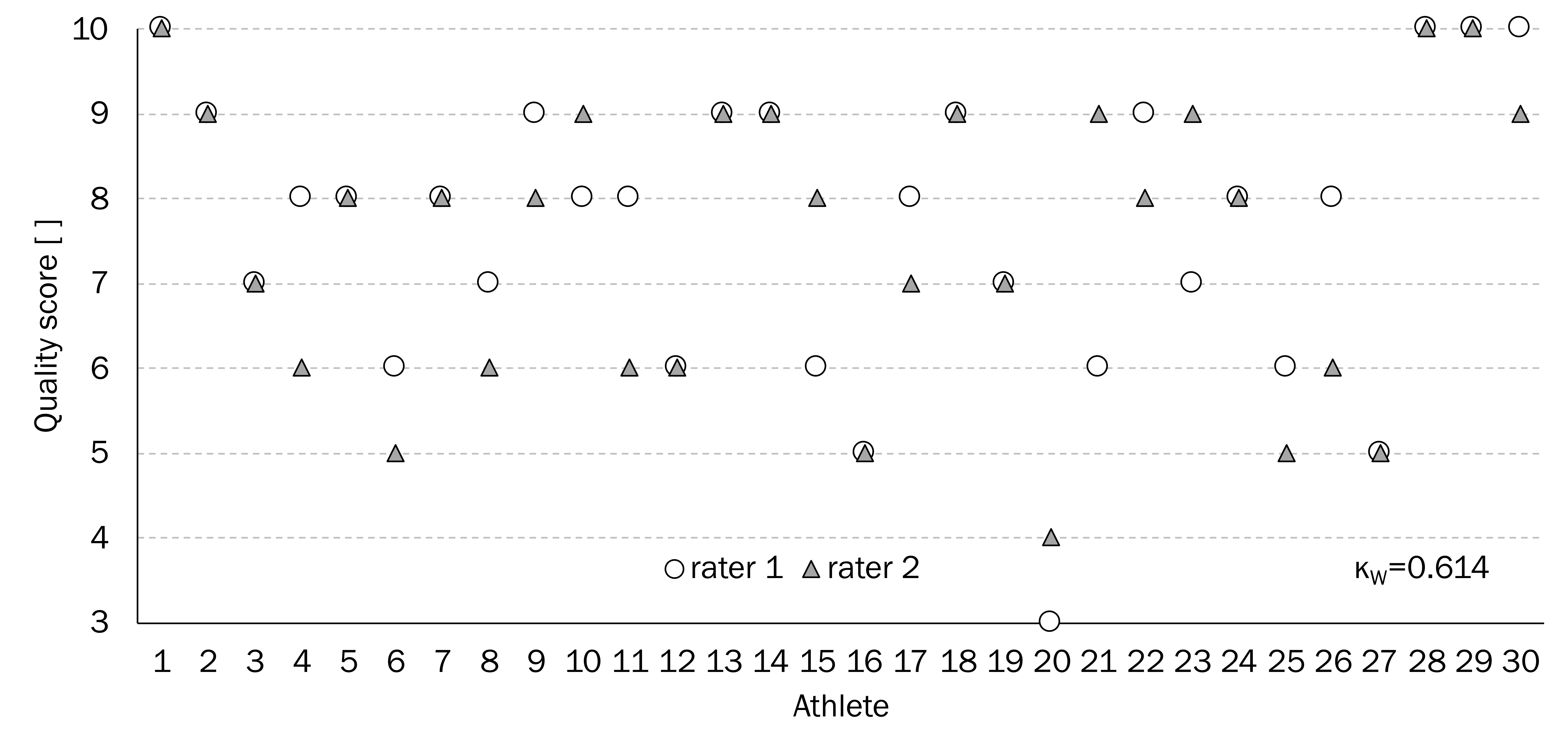

Supplement: Supplementary file 4 — Additional file 4: Figure 4S. Comparison of raters’ judgements of movement quality of the Balance 90° rotation hop (sum of both sides). [file 13102_2024_886_MOESM4_ESM.png]
